# Gut microbiome of treatment-naïve MS patients of different ethnicities early in disease course

**DOI:** 10.1038/s41598-019-52894-z

**Published:** 2019-11-08

**Authors:** R. E. Ventura, T. Iizumi, T. Battaglia, Menghan Liu, G. I. Perez-Perez, J. Herbert, M. J. Blaser

**Affiliations:** 1 Department of Medicine, New York University School of Medicine, New York, NY USA; 2 Department of Microbiology, New York University School of Medicine, New York, NY USA; 3 Center for Advanced Biotechnology and Medicine, Rutgers University, Piscataway, NJ USA; 4 Department of Neurology, New York University School of Medicine, New York, NY USA

**Keywords:** Multiple sclerosis, Microbiome, Multiple sclerosis, Neuroimmunology

## Abstract

Although the intestinal microbiome has been increasingly implicated in autoimmune diseases, much is unknown about its roles in Multiple Sclerosis (MS). Our aim was to compare the microbiome between treatment-naïve MS subjects early in their disease course and controls, and between Caucasian (CA), Hispanic (HA), and African American (AA) MS subjects. From fecal samples, we performed 16S rRNA V4 sequencing and analysis from 45 MS subjects (15 CA, 16 HA, 14 AA) and 44 matched healthy controls, and whole metagenomic shotgun sequencing from 24 MS subjects (all newly diagnosed, treatment-naïve, and steroid-free) and 24 controls. In all three ethnic groups, there was an increased relative abundance of the same single genus, *Clostridium*, compared to ethnicity-matched controls. Analysis of microbiota networks showed significant changes in the network characteristics between combined MS cohorts and controls, suggesting global differences not restricted to individual taxa. Metagenomic analysis revealed significant enrichment of individual species within *Clostridia* as well as particular functional pathways in the MS subjects. The increased relative abundance of *Clostridia* in all three early MS cohorts compared to controls provides candidate taxa for further study as biomarkers or as etiologic agents in MS.

## Introduction

Evidence has been emerging for a role of alterations in the gut microbiome in several autoimmune diseases including MS^1–8^, presumably through interactions between the intestinal microbiome and the host immune system. Mouse studies have found that microbiota shape the gut-associated lymphoid tissue, with particular bacteria supporting differentiation of pro-inflammatory T-cells and others leading to anti-inflammatory responses^9–11^. Mice raised in germ free conditions are resistant to the development of experimental autoimmune encephalitis (EAE), an MS model disease, with susceptibility restored when commensal microbiota are introduced^12^.

Given that genetic factors are believed to have only modest effects on susceptibility and severity of MS^13^, it is important to consider environmental mediators such as the microbiome. To capture any potential effect of the microbiome on MS disease initiation, it is ideal to examine early in the disease course and to avoid subjects that have received disease-modifying agents (DMTs), since disease drift over time^14^, comorbid conditions such as constipation^15^, and exposure to DMTs^1,16,17^ could affect microbiome composition. However, prior studies of the microbiome in adult MS subjects have mostly included subjects with current or prior disease-modifying therapies and/or with relatively long disease durations^1–5,16,18^.

There are known differences in MS disease severity between patients of different ethnicities but the reasons for this have not not been determined^19–22^. Differences in the microbiome could potentially lead to differences in disease severity, and given that the microbiome may segregate across ethnic populations^23,24^, we sought to separately analyze the microbiota in members of three major ethnic groups in the populations we serve: African Americans (AA), Hispanics (HA), and Caucasians (CA). In this study, we evaluated the gut microbiota in subjects who were naïve to disease-modifying treatments, most of whom had newly diagnosed MS, in each of the three ethnic groups. Using two different analytical approaches, we identified a single taxon associated with MS cases across all three ethnic groups.

## Methods

### Study population

Institutional Review Board approval from New York University Langone Medical Center was obtained and all subjects provided written informed consent. All methods were performed in accordance with the relevant guidelines and regulations. Relapsing-remitting AA, HA, and CA MS subjects as defined by the 2010 McDonald Criteria^25^ were recruited from the NYU Multiple Sclerosis Comprehensive Care Center clinic population. Ethnicity matched healthy controls also were recruited. About half of the controls were family members of the cases, and the remainder responded to advertisements around the NYU Medical Center campus. All cases and controls were recruited in the same geographic region. Ethnicity was determined based on self-identification and a survey of geographic origin. Subjects of mixed ethnicity, e.g., Caribbean African Americans, were not included. All enrollees were between the ages of 18 and 70. The only allowable medications at study entry were NSAIDs and analgesics at FDA-approved doses. Exclusion criteria included current or prior use of disease-modifying or biologic therapies for MS, recent (<3 months prior) use of any antibiotic therapy, current extreme diet such as parenteral nutrition, known inflammatory bowel disease, any GI tract surgery leaving permanent residua such as gastrectomy, bariatric surgery, or colectomy, or currently active malignancy. A dietary survey, modified from that described by Matijasic and others^26^, was administered to subjects. This survey assessed general diet types and durations (eg, vegetarian, gluten free, paleo, etc), as well as a current weekly estimate of consumption of a variety of foods such as yoghurt, probiotics, red meats, white meat, breads, fatty foods, and fruits and vegetables.

### Stool collection

Subjects were provided with stool collection containers as well as coolers with ice packs. Once the sample was collected and placed in the coolers, the sample was either picked up by courier or brought into the clinic within 24 hours of production. The samples were then transferred to the NYU Biorepository and stored at −80 °C until DNA extraction.

### DNA extraction

DNA was extracted from an ~20 mg aliquot of feces using the PowerSoil DNA Isolation Kit (MOBIO, West Carlsbad CA), according to the manufacturer’s protocol. The concentration of extracted DNA was determined by Nanodrop 1000 (Thermo Scientific, Watham MA), and DNA was stored at −20 °C until used.

### Library preparation for high-throughput sequencing

All samples were PCR-amplified and barcoded for multiplex High-Throughput Sequencing (HTS), using primers targeted to the V4 region of the bacterial 16S rRNA gene under uniform PCR conditions that included 3 min at 94 °C and 45 cycles of 45 s at 94 °C, 60 s at 50 °C, and 90 s at 72 °C with final extension for 10 min at 72 °C [1]. We used forward primer (AAT GAT ACG GCG ACC ACC GAG ATC TAC ACT ATG GTA ATT GTG TGC CAG CMG CCG CGG TAA) that includes a 5’ Illumina adaptor, forward primer pad, 2 bp linker and the 515 F 16 S rRNA primer, and reverse primer (CAA GCA GAA GAC GGC ATA CGA GAT NNNNNNNNNNNN –AGT CAG TCA G-CC-GGA CTA CHV GGG TWT CTA AT) that includes the Illumina 3’ adapter with 12-nt error-correcting Golay barcode, reverse primer pad, 2 bp linker and the 806 R 16 S rRNA primer. We ran PCRs in triplicate using 0.2 µM of the primers, 1 µl of template and 1X HotMasterMix (5 PRIME, Gaithersburg MD), and cleaned the products using a PCR Purification Kit (Qiagen) after pooling. Cleaned PCR products were quantified using the Qubit dsDNA HS Assay Kit (Invitrogen™, Eugene OR), then adjusted to an optimal molarity as described. Sequencing was performed using the Illumina MiSeq platform in the NYULMC Genome Technology Core.

### Taxonomic and ecological analyses

We analyzed all sequence data using the QIIME software package (version Mac Qiime 1.8.0)^27^, essentially as described^28^. After filtering procedures, similar sequences were clustered into operational taxonomic units (OTUs) using an open reference approach with UCLUST^29^ against the Greengenes Core set. A representative sequence was then aligned using PyNAST, and FastTree created phylogenetic trees. Rarefaction analysis used Chao-1 and whole PD to measure α-diversity. Unweighted UniFrac distances were calculated to assess β-diversity; the Unweighted paired group method with arithmetic mean (UPGMA) was performed for UniFrac-based jackknifed hierarchical clustering. Principal coordinates analysis (PCoA) of UniFrac distance matrices provided graphical representation using KiNG. ANOVA was used to compare OTU and genus-level abundances, and Linear discriminant analysis (LDA) effect size (LEfSe), a tool that can compare differences of relative abundance between ≥2 biological conditions^30^, also was used for analysis.

### Whole metagenome sequencing

Fecal samples were used for Whole metagenome sequencing (WMS) using the Illumina HiSeq. 4000 platform, with 150-bp paired-end reads (2 × 150), across 4 lanes. Quality filtering was performed by removing host-contaminated sequences using KneadData, and applying a conservative quality threshold criteria of Phred score >20 and minimum sequence length >105 bp, using Cutadapt^31^. Following quality filtering, the marker-based tool, MetaPhlan2 (v.2.6.0)^32^ was used for profiling the composition of microbial communities. The biomarker identification tool, LEfSe^33^ also was used to identify enrichment of species between 2 or more groups. Additionally, filtered reads were processed using the microbial functional profiling tool, HUMAnN2 (v0.11.1)^34^. In short, reads were initially aligned against a sample-specific microbial pangenome database (ChocoPhlAn) from species detected using MetaPhlan2. Any unaligned reads were subjected to a translated search against the recommended UniRef90 protein database, and MetaCyc pathways were reconstructed. Abundance tables were reported by default to RPK (Reads Per Kilobase of transcript) and further normalized to CoPM (Copies per Million) to account for differences in gene length and sampling depth. The WHAM! program^35^ was used for the analysis of metagenomic data.

### Microbiome network analysis

Network was constructed at genus level by summing up all associated OTUs for each genus. Species that were present in at least 30% of the samples were selected for network inference by SPIEC-EASI [42]. The default setting of SPIEC-EASI accepts absolute abundance of taxa as input and applies centered log-ration transformation to eliminate the unit-sum constraint of data [86]. Networks were constructed with the SPIEC-EASI [42] package in R in neighborhood selection mode with parameters set as *method* = *“mb,” sel.criterion* = *“bstars,” lambda.min.ratio* = *0.0*2*−1, nlambda* = *100, pulsar.params* = *list(rep.num* = *20, ncores* = *2*). The edge directions were predicted based on the sign of mean coefficients in beta matrix. The methods for edge centrality and betweenness are calculated as described^36^.

## Results

### Subject demographics

We enrolled a total of 45 patients with MS from the following ethnic groups: Caucasian Americans [CA (15)], Hispanic Americans [HA (16)], and African Americans [AA (14)] (Table 1). We enrolled 45 controls equally distributed across the three ethnic groups but the sample from one AA control could not be sequenced due to poor quality. The patients in the MS group as a whole and in particular the CA MS group were significantly older than the control groups. There were no significant differences between any of the groups in sex distribution. All but three MS subjects provided a sample within 6 months of diagnosis. The 3 subjects who had stool specimens obtained more than 6 months after diagnosis were all AA and had been diagnosed 2, 7, and 8 years prior to the time of stool collection. 84% of the subjects had stool collection within 1 year of symptom onset. There were no differences between the MS groups in terms of steroid or probiotic use. A dietary questionnaire was administered and there was no significant difference between controls and MS subjects in terms of vegetarian/vegan/Mediterranean diets or average weekly consumption of fruit/vegetables, red meat, or bread slices (data not shown).

**Table 1:** Demographic features of the 89 subjects included in the intestinal microbiota sequencing analysis.

	Caucasian		Hispanic		African American		All	
	Control	MS	Control	MS	Control	MS	Control	MS
Number of samples	15	15	15	16	14	14	44	45
Age^&^ [years; mean ± SD (range)]	28.0 ± 8.5 (21–55)	39.9 ± 12.3 (22–59)^*^	32.7 ± 6.8 (20–44)	32.8 ± 10.5 (19–56)	34.8 ± 10.5 (35–58)	39.1 ± 14.7 (22–63)	31.8 ± 9.0 (20–58)	37.1 ± 12.7 (19–63)^$^
% Female	60	85	53	69	77	77	64	76
% within 6 months of diagnosis	/	100	/	100	/	77	/	93
% within 1 year of symptom onset		100		94		57		84
% taking Probiotics	7	27	47	38	29	21	27	29
% receiving steroids in prior month	/	13	/	0	/	14	/	9

^$^p = 0.02 versus all controls; *p = 0.005 versus CA control; ^&^age at stool collection.

### Differences in intestinal microbial composition

**Figure 1:**
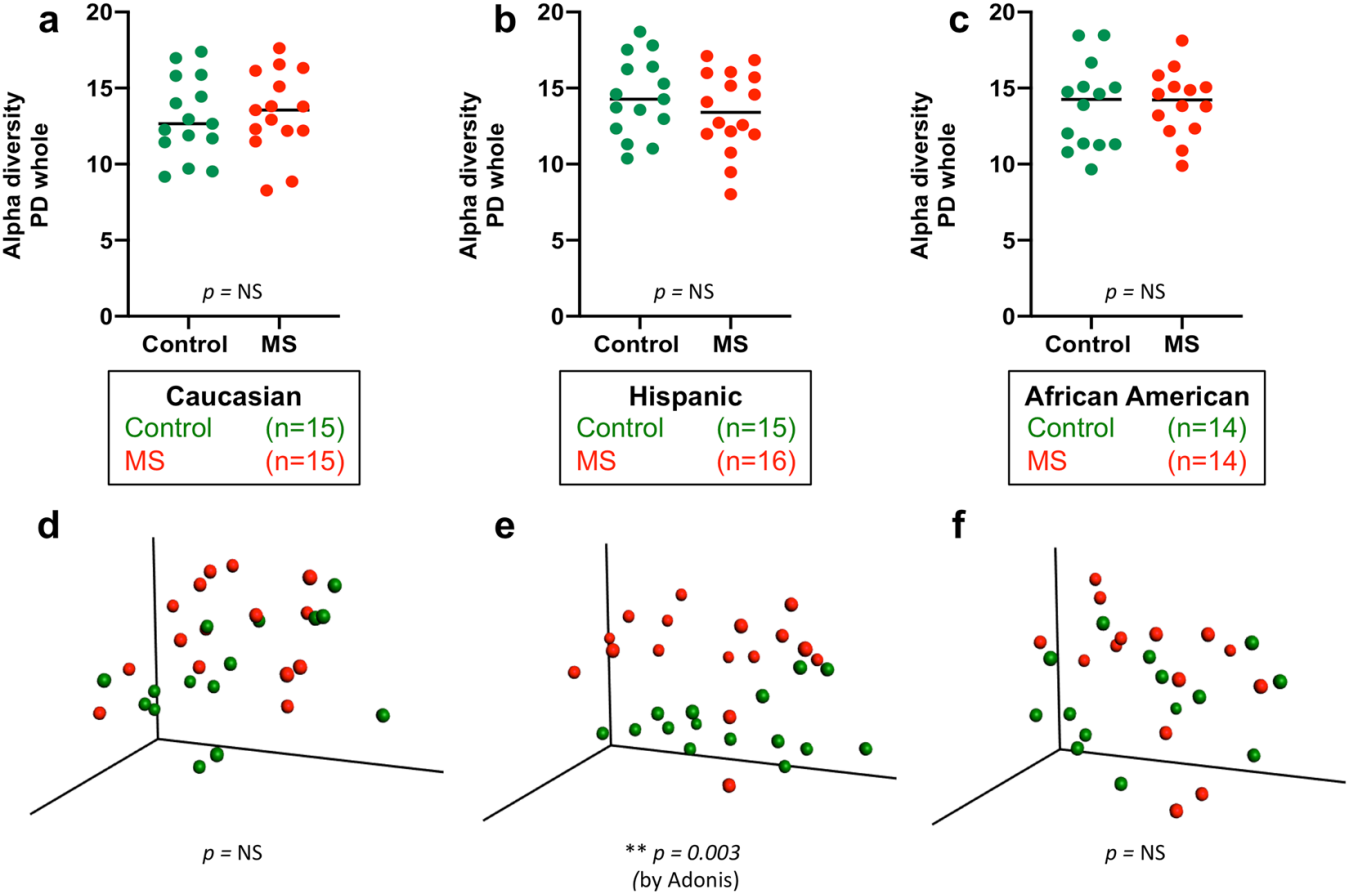
Community characteristics of the fecal microbiota from 45 MS patients and 44 Control subjects. Shown are representations of Alpha-diversity, based on Phylogenetic Distance score (Panels a–c), and PCoA plot of Beta-diversity, based on Unweighted UniFrac analysis (Panels d–f), sorted by ethnicity.

We compared the community richness and structure of the fecal microbiota of the different clinical groups by assessing Alpha- and Beta-diversity. Alpha-diversity provides a measure of the variety of species present in a sample, and Beta-diversity measures differences in the composition of the microbial communities between samples^37^. There were no significant differences in Alpha- or Beta-diversity between the three ethnic groups in either the MS subjects or the control subjects (Fig. S1). There were no significant differences in Alpha-diversity between any of the three ethnic groups and their respective controls, and there were no significant differences in Beta-diversity between the MS and control subjects in the CA or AA ethnicities (Fig. 1). However, there were significant differences in Beta-diversity between MS and control in the Hispanic-American subjects (p = 0.003).

Given that there were differences in age between the MS and control populations studied, we next assessed whether there were any substantial microbiome composition differences between the younger and older subjects. We found no significant differences in Alpha-diversity in those <30 or ≥30 in either the MS or control subjects (data not shown). There also were no significant differences in Beta-diversity in control subjects <30 or ≥30, but among MS subjects, there was a weakly significant difference (p = 0.04) in Beta-diversity between those <30 or ≥30 (Fig. S2). There also was a difference in Beta-diversity between control and MS subjects <30 (p = 0.03), but not between control and MS subjects ≥30. Of the 90 subjects, about half were regular consumers of yoghurt, and 25 (28%) used probiotics (Table 1). There were no significant differences in Alpha- or Beta-diversity in MS subjects or controls when we assessed subgroups who had or had not consumed yoghurt or probiotics at least once per week, except that Beta-diversity differed between MS and control subjects (p = 0.003) in those not using any probiotics (Fig. S3).

**Figure 2:**
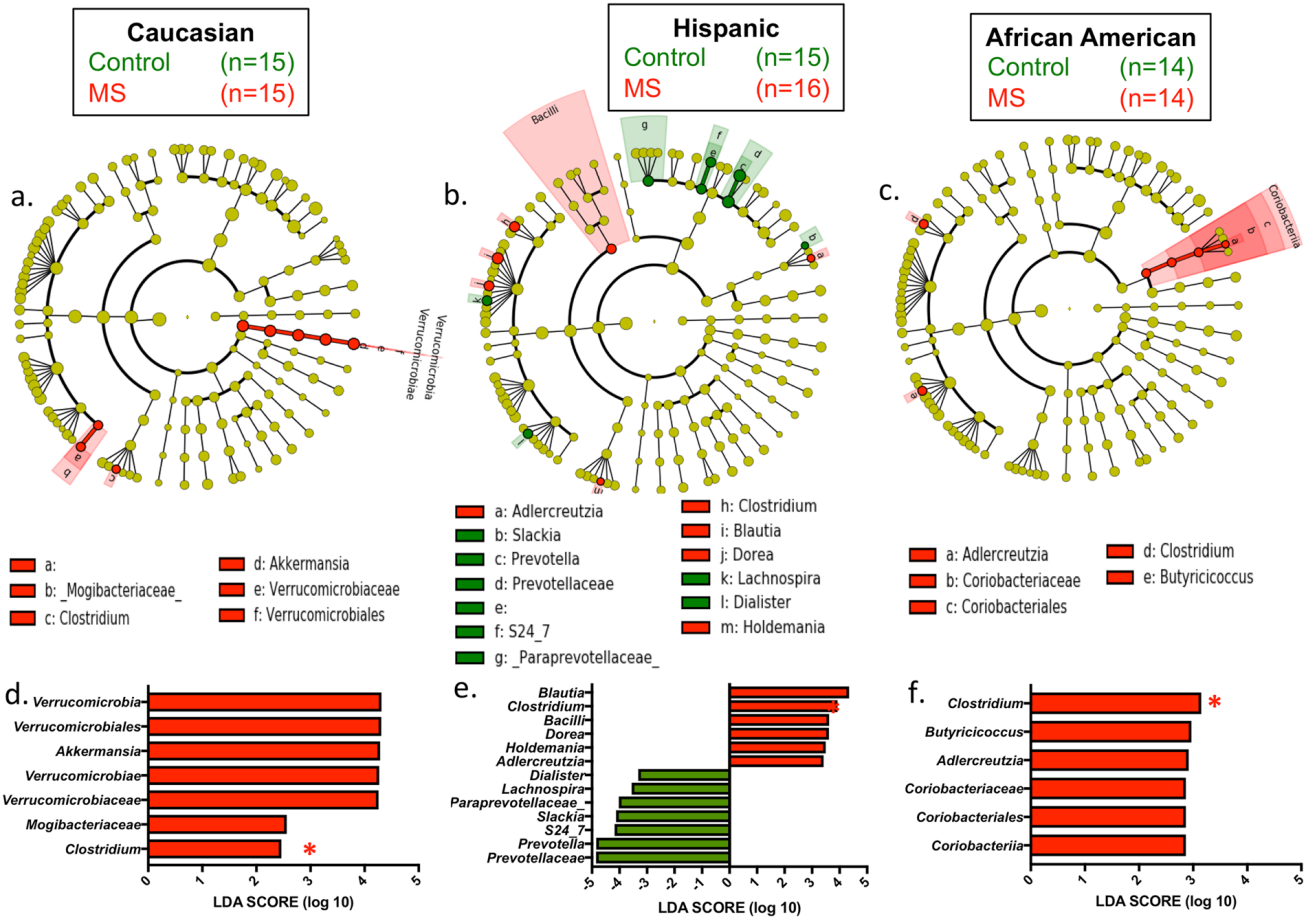
Differential representation of taxa in the MS and control subjects. Cladogram (**a–c**) and linear discriminant analysis (LDA) score (**d–f**) of differences by LDA effect size (LEfSe) in each of the three ethnic groups between the MS and control samples. *Clostridium* species (red star in d**–**f) were significantly enriched in MS patients in all three ethnic groups.

All three MS ethnic groups had an increase in the relative abundances of *Clostridia* species compared with their respective ethnicity-matched controls (Fig. 2). For other taxa, we found significant differences between cases and controls in one or two ethnic groups but not in all three; *Clostridia* was the singular exception. There was a broad range of relative abundances of the *Clostridia* amongst the subjects (Fig. S4), but in each of the three ethnic groups, the median was higher in the MS patients than in the control subjects. The other taxa showing significant differences include: (i) in Caucasian MS versus controls, there was an increase in the phylum *Verrucomicrobiales* and in *Akkermansia* at the genus level; (ii) HA MS and AA MS but not CA MS had an increase in *Adlercreutzia* versus their respective controls; (iii) HA MS also had an increase in *Blautia*, *Holdemania*, and *Dorea*, and a decrease in *Prevotella*, Slackia, *Lachnospira*, and *Dialister*; and (iv) AA MS had an increase in *Butyricococcus*.

**Figure 3:**
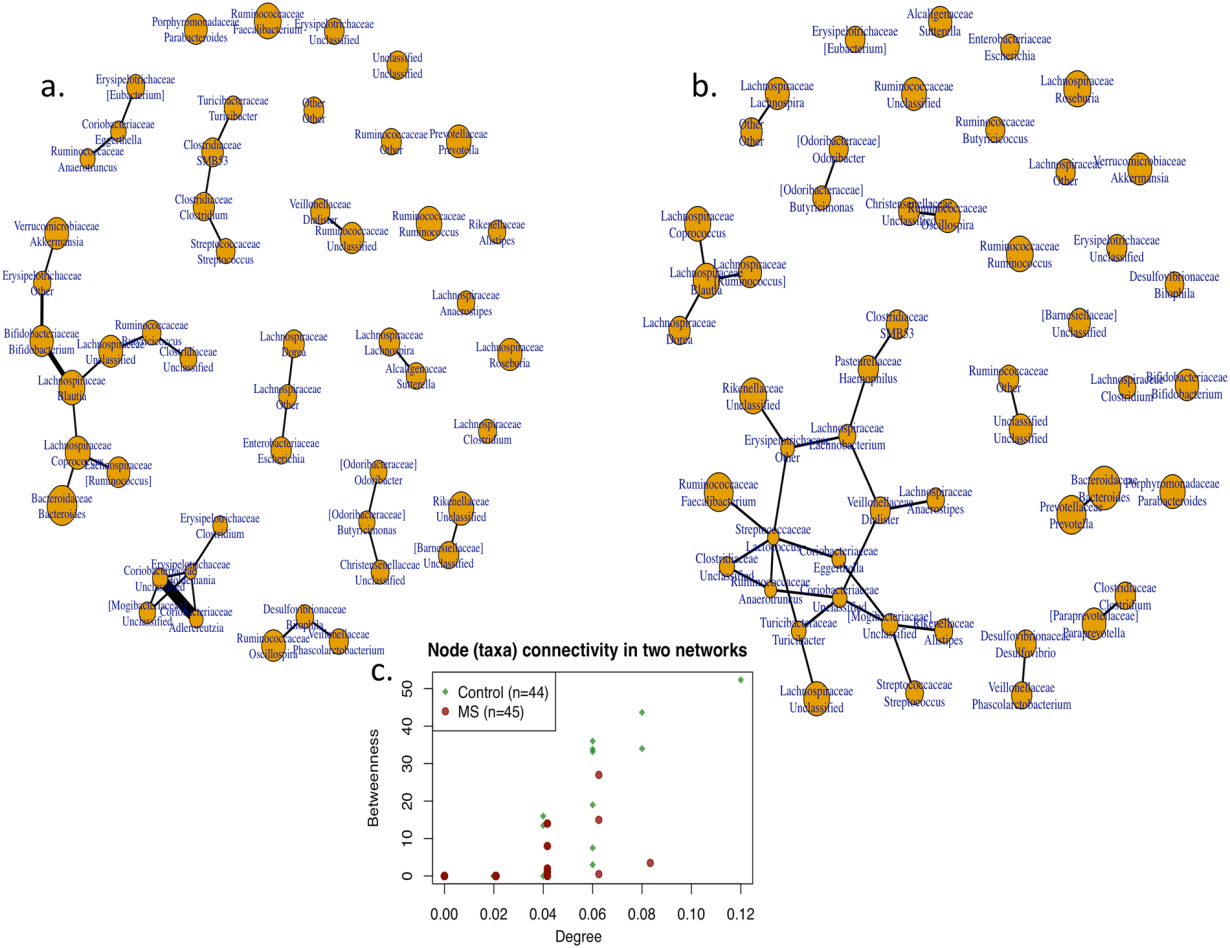
Network analysis of fecal samples from MS patients and controls, based on 16S rRNA taxonomic representation. Distribution of sub-networks in MS patients (Panel A) and Controls (Panel B), and comparison of node (taxa) connectivity in the microbial networks (Panel C). There was significantly lower connectivity in samples from MS patients than from controls, in two-sided tests of degree (p = 0.02), with borderline differences in betweenness (p = 0.09) network characteristics.

Since it may be the case that more than one change in bacterial taxa is associated with a pathogenic state such as MS, we performed a network analysis to determine whether there were MS-specific differences in pairs or groups of co-occurring bacteria. This analysis showed that there was a decrease in connectivity in the structure of the sub-networks in the MS subjects compared to the controls, with a significant difference in the degree characteristic (p = 0.02), and a borderline difference in betweenness (p = 0.09) (Fig. 3). These findings support the hypothesis of global compositional differences in MS patients compared with controls.

### Whole metagenomic shotgun sequencing

**Figure 4:**
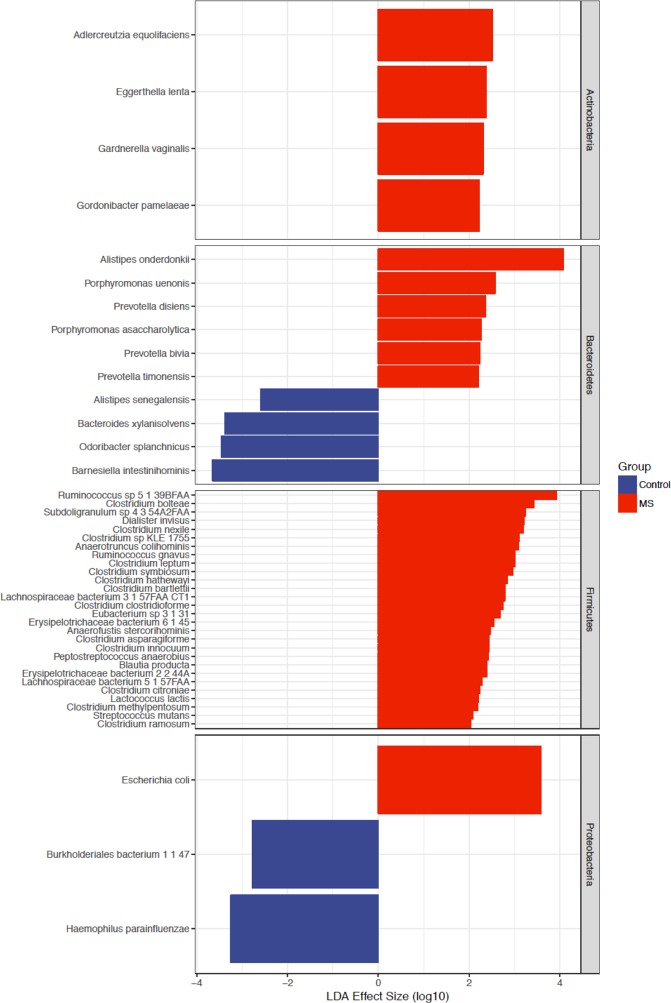
Differential representation of taxa in the MS and control subjects from Whole Genome Sequencing (WGS) analysis. Results are shown for the 24 samples each from the MS patients and the control subjects, as determined by LDA effect size (LEfSe). The differential representation of significance for Actinobacteria and Firmicutes is asymmetric between MS and control subjects.

**Figure 5:**
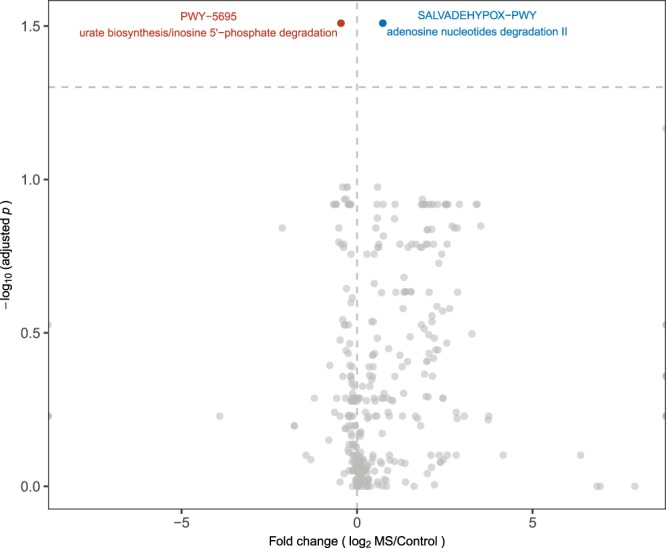
Volcano plot of 296 pathways in the intestinal metagenome of MS patients and controls. Data are from high throughput sequencing of 48 fecal specimens (24 each from MS patients and controls), with analysis at mean sequence depth of 6.1 GB, and <1% host contamination. Each circle represents one pathway. Fold change was calculated using the average measurements for control compared to MS subjects. Significance was determined using Wilcoxon rank-sum tests with a false discovery rate (FDR) adjustment. The dashed horizontal line represents p = 0.05. The pathways that are significantly differentially abundant between MS and control subjects are indicated in blue (over-represented in MS, n = 4) or red (under-represented in MS, n = 2).

**Figure 6:**
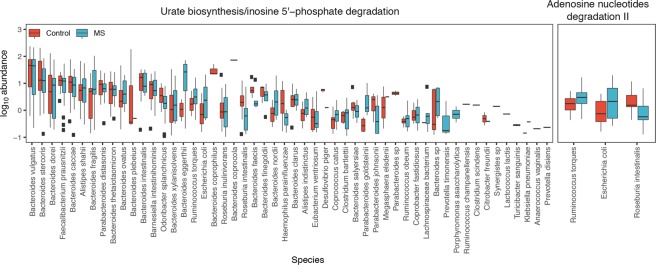
Bacterial taxa with functional contributions to the four pathways that are significantly under-represented in MS patients. The identified taxa are shown at the species level. Boxplots display the mean, first and third quartiles. Black dots represent outliers, with values > 1.5 times the interquartile range. Taxa are ordered by the total pathway abundance across all subjects. See Fig. 5.

To provide another view of the species present in the MS patients and control subjects and to evaluate differences in gene functions, we performed whole metagenomic sequence analysis on 24 of the MS subjects versus 24 controls, essentially equally distributed across ethnic groups. All of the subjects selected for this analysis were treatment-naïve, had not received steroids for the prior month and were not taking probiotics, and all samples had been obtained within 6 months of the initial MS diagnosis (Table 2). Among the original control sample of 44 subjects, the 24 controls for this subanalysis were selected based on the criteria of no probiotics (or steroids) and then were ethnicity and age-matched to the MS subjects to the degree possible. After eliminating poor quality reads, this analysis yielded a mean sequence depth of 6.1 ± 0.8 Gbp per sample, with host gene contamination <1%. At a taxonomic level, 6 species were significantly enriched in the control subjects vs 41 in the MS subjects; the latter included 13 individual *Clostridia* species (Fig. 4). Several species also were significantly decreased in the MS subjects, including *Bacteroides xylanisolvens*. Analysis of 296 functional pathways identified from the metagenomic data demonstrated significant abundance elevations of two pathways and decreases in four pathways (adjusted p value < 0.05) between the MS and control subjects (Fig. 5). The taxa that significantly contributed to the differential abundances of these six pathways were relatively limited (Fig. 6).

**Table 2:** Demographic features of the 48 subjects included in the shotgun metagenomic sequencing analysis.

	Caucasian		Hispanic		African American		All	
	Control	MS	Control	MS	Control	MS	Control	MS
Number of samples	9	9	8	8	7	7	24	24
Age [years; mean ± SD (range)]	30.3 ± 10.2 (22–55)	42.0 ± 12.6 (22–59)^*^	33.1 ± 8.1 (20–44)	31.6 ± 9.6 (19–50)	37.8 ± 11.9 (27–58)	36.4 ± 12.4 (23–54)	33.5 ± 10.1 (22–55)	36.9 ± 12.0 (22–58)
% Female	67	78	50	75	71	71	63	75

All MS subjects selected for these analyses were treatment-naïve, were diagnosed within 6 months of the specimen collection, had symptom onset within the prior year, and did not receive steroids in the prior month. The MS CA subjects were significantly (*p = 0.05) older than the Control CA subjects; there were no significant differences in the other pairwise comparisons by age or sex.

## Discussion

Our study evaluated differences in the gut microbiota in newly diagnosed, DMT-naive MS subjects in three ethnic groups in relation to their matched controls. We chose this study design to minimize variation in comparisons across the study groups and to avoid the confounding effects of both long-standing disease and its treatment. Analysis based on 16S rRNA sequencing showed increased representation of *Clostridia* in each of the groups compared to their respective control groups, and these differences were confirmed using metagenomic shotgun sequencing in specimens from a subset of recent-onset, treatment-naïve MS subjects. The consistency of the finding across the three ethnic groups as well as the separate 16S and metagenomic findings increase our confidence in the observation.

Unlike our cohort, prior studies on the microbiome in MS patients mostly included subjects who were currently receiving or had been exposed to disease modifying treatments and who had considerable disease durations (average ranges from 7–13 years for four prior studies^1–4^), which may explain some of the differences in the findings. In another recent study, for example, disease durations are not mentioned, but the onset for the MS subjects ranged from 1983 and 2016, with only 14/71 MS subjects having onset between 2014–2016^5^. Patients with longer disease duration tend to have higher rates of constipation^38^; differences in intestinal transit time may lead to microbiome compositional changes, including increased representation of *Methanobrevibacter* species^1,15^. The microbiome also changes with age^39^ and differs by geographic location of subjects^40^, both of which may also account for differences in study findings. Differences across studies could also reflect technical factors, such as variation in DNA extraction and sequencing methodologies.

Our work complements a recent report in which spore-forming bacteria were specifically isolated from 25 MS patients not currently on DMTs versus controls, and *Clostridia* were found to be significantly enriched in the MS population versus controls^18^. In that study, the average disease duration was 13.5 years; as with the above studies, the long disease duration and concomitant constipation could alter the composition of the microbiome, with constipation itself associated with increased *Clostridia*^41^. In our study of newly diagnosed MS subjects, we also find increased *Clostridia* which is consistent with a potential role in MS pathogenesis. Cekanaviciute and colleagues found that the spore-forming bacteria from MS subjects (versus those from controls) impaired the *in vitro* differentiation of IL-10-secreting, regulatory T lymphocytes, consistent with a pathogenic role; however, in a small sub-study, neither the spore-forming bacteria from 2 MS or 2 control subjects had an exacerbating impact on EAE induction in antibiotic-fed mice^18^. These negative findings could reflect small sample size and variability in gut biota, the lack of other bacterial species that are needed in a network to exacerbate disease, changes in the spore-forming bacteria present in long-standing MS, or that any protective effect relates to different pathophysiologic steps. Further work is needed to elucidate a functional role for the increase in *Clostridia* representation that we and others have observed in the MS population.

Prior studies had conflicting reports on Clostridial abundance when the whole gut microbiome was compared between MS patients and controls. While one other prior study had findings consistent with ours using OTU abundance data (although it lacked a species-level analysis)^2^, several other studies found a decrease of *Clostridia* species in the gut microbiome of MS subjects compared to healthy controls. For example, in comparison to controls, studies have found a decrease in *Clostridium perfringens* Type A in MS subjects^42^, or a decrease in *Clostridia* clusters XIVa and IV in MS subjects^3^. Nevertheless, the genus Clostridium is highly diverse^43,44^, and species and strains might have opposing host interactions^45^.

Two reports found differences in particular *Clostridium* OTUs comparing treated versus untreated MS subjects, but no significant differences when comparing all MS patients versus controls^1,16^. In addition, disease modifying drugs that are taken orally may inhibit the growth of *Clostridium* species *in vitro*, suggesting that the antibacterial properties of these agents could alter the microbiome^46^.

The findings in the CA subjects in our study most closely matched the data from Jangi *et al*., which may reflect that 97% of those MS subjects were Caucasian^1^; both studies showed increased *Akkermansia* abundance versus controls. Another study found changes that more closely matched the findings in our HA population, with increases in *Blautia* and *Dorea*, and decreases in *Prevotella*^2^, but the ethnic distribution of the subjects was not reported.

On average, AA and HA patients have higher relative disability than CA patients with MS^21^, with AA patients having an earlier time to increased disability despite earlier diagnosis after symptom onset^19,20^. This relationship has been found to be independent of socioeconomic factors including insurance type^21^. Whether or not the differences we found in the microbiota compositions between our CA, HA, and AA groups of MS patients may account for some of the differences in disease course between ethnic groups requires further study.

Limitations of our study include relatively small patient group size, differences in age between some of the cohorts, and lack of BMI and other metabolic data. While we excluded subjects with any history of DMTs or a history of steroids for the 3 months prior to stool collection, it is possible there were other remote drug exposures that could have altered the microbiomes. Although we have found the same significant differences between all MS subgroups and controls, it is not certain whether the changes identified are causal for MS, result from MS, or are incidental (falsely-positive). Although further taxonomic and metagenomic characterization of the intestinal microbiota in early MS cases will be necessary, this work provides potential biomarkers for future analyses. Further studies will help determine whether candidate species alter immune functions to contribute to early MS, which would have implications for diagnosis, prevention, and treatment of MS.

## Supplementary information

Supplemental Figures

## Data Availability

Any data not published within the article will be shared as an anonymized dataset upon request from any qualified investigator. All metagenomes have been deposited in the European Nucleotide Archive (ENA) and are available at the European Bioinformatics Institute (EBI) under the accession number PRJEB28543 (https://www.ebi.ac.uk/ena/data/view/PRJEB28543).
