# Supplementary material for: Gut microbiome of treatment-naïve MS patients of different ethnicities early in disease course
Source: Sci Rep. 2019 Nov 8;9:16396. doi: 10.1038/s41598-019-52894-z (PMC6841666; doi:10.1038/s41598-019-52894-z)
Supplement: Supplementary file 1 — Supplemental Figures [file 41598_2019_52894_MOESM1_ESM.pdf]

Gut microbiome of treatment-naïve MS patients of different ethnicities early in disease course

R.E. Ventura, MD PhD[*], T. Iizumi, MD PhD[ac], T. Battaglia[a], Menghan Liu[a], G.I. Perez-Perez, DSc[a], J. Herbert, MD[+], M.J. Blaser, MD[abc]
[a]Department of Medicine, New York University School of Medicine, New York, NY USA
[b]Department of Microbiology, New York University School of Medicine, New York, NY USA
[c]Center for Advanced Biotechnology and Medicine, Rutgers University, Piscataway, NJ USA
+Deceased

Corresponding Author:
Rachel Ventura, MD, PhD
rachel.ventura@gmail.com
220 Riverside Boulevard Apt 14M
New York, NY 10069
Telephone: 917-312-2160

Figure Legends

Figure S1. Community characteristics of the fecal microbiota from 45 MS patients and 44 control subjects, comparing the three ethnic groups to one another. Shown are representations of Alpha-diversity, based on Phylogenetic Distance score (Panels a,b), and PCoA plot of Beta-diversity, based on Unweighted UniFrac analysis (Panels c,d).

Figure S2. Community characteristics of the fecal microbiota from the MS patients and control subjects, comparing those at or above versus below age 30. Shown is a representation of Alpha-diversity, based on Phylogenetic Distance score (Panel a), and PCoA plots of Beta-diversity, based on Unweighted UniFrac analysis (Panels b-e). By Adonis testing, there were significant differences in Beta-diversity among MS subjects by age (*p=0.04), and in those under 30, between the MS patients and the controls (*p=0.03).

Figure S3. Community characteristics of the fecal microbiota from the MS patients and control subjects, stratified by probiotic usage. Shown are representations of Alpha-diversity based on Phylogenetic Distance score (Panel a) and PCoA plots of Beta-diversity, based on the Unweighted UniFrac analysis (Panels b-e).
By Adonis testing, there was a significant difference in Beta-diversity between MS and control subjects who were not taking probiotics (*p=0.03).

Figure S4. Relative abundances of *Clostridium* species in the fecal samples, according to clinical status and ethnicity. Data are shown as Medians + Interquartal Range (IQR) and 95% Confidence Intervals in box and whiskers plots. Differences between MS cases and controls were significant in all three ethnic groups, by LEfSe analysis (see **Figure 2**).

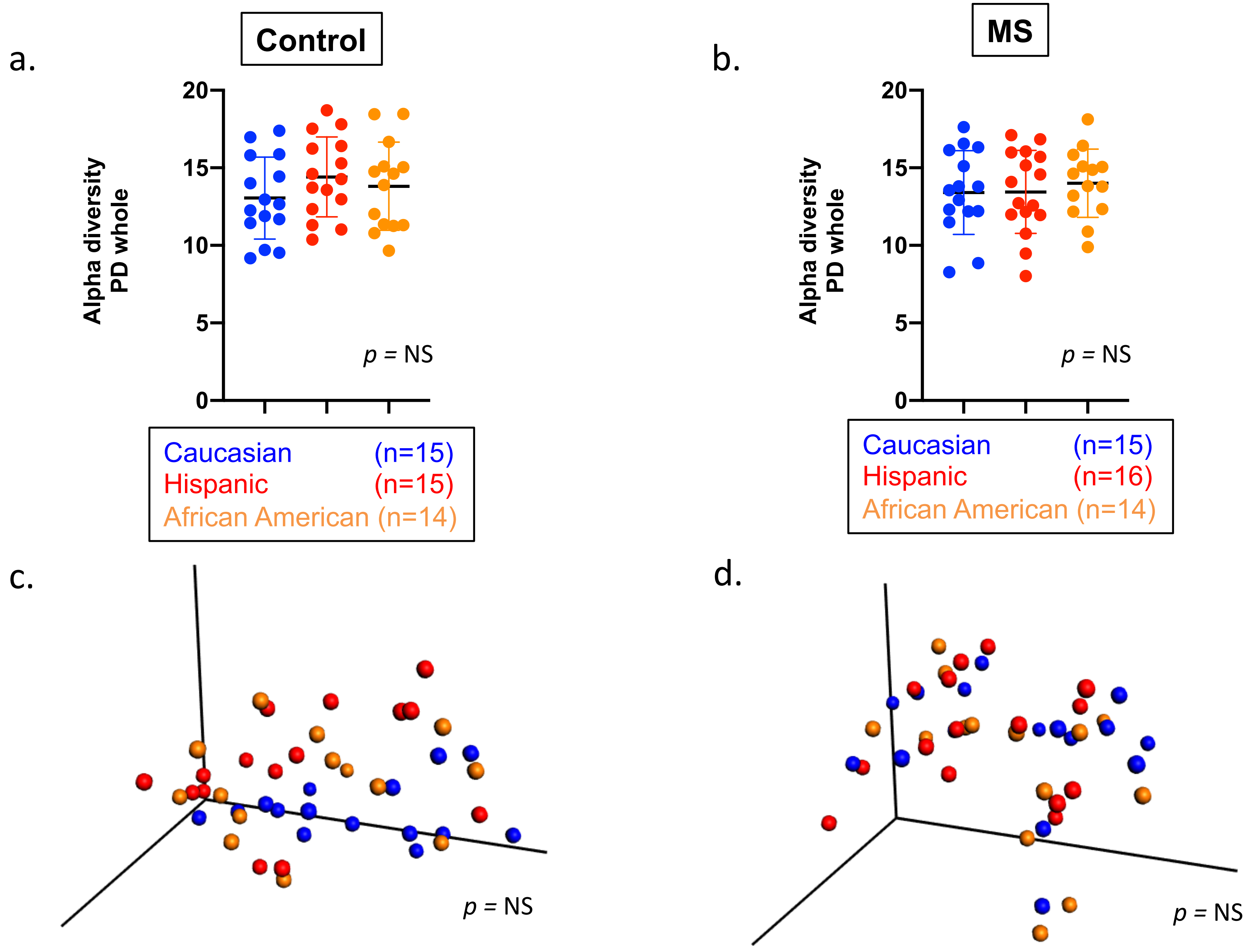
a.
Control
Alpha diversity PD whole
20
15
10
5
0
p = NS
Caucasian (n=15)
Hispanic (n=15)
African American (n=14)
b.
MS
Alpha diversity PD whole
20
15
10
5
0
p = NS
Caucasian (n=15)
Hispanic (n=16)
African American (n=14)
c.
p = NS
d.
p = NS

Figure S1.

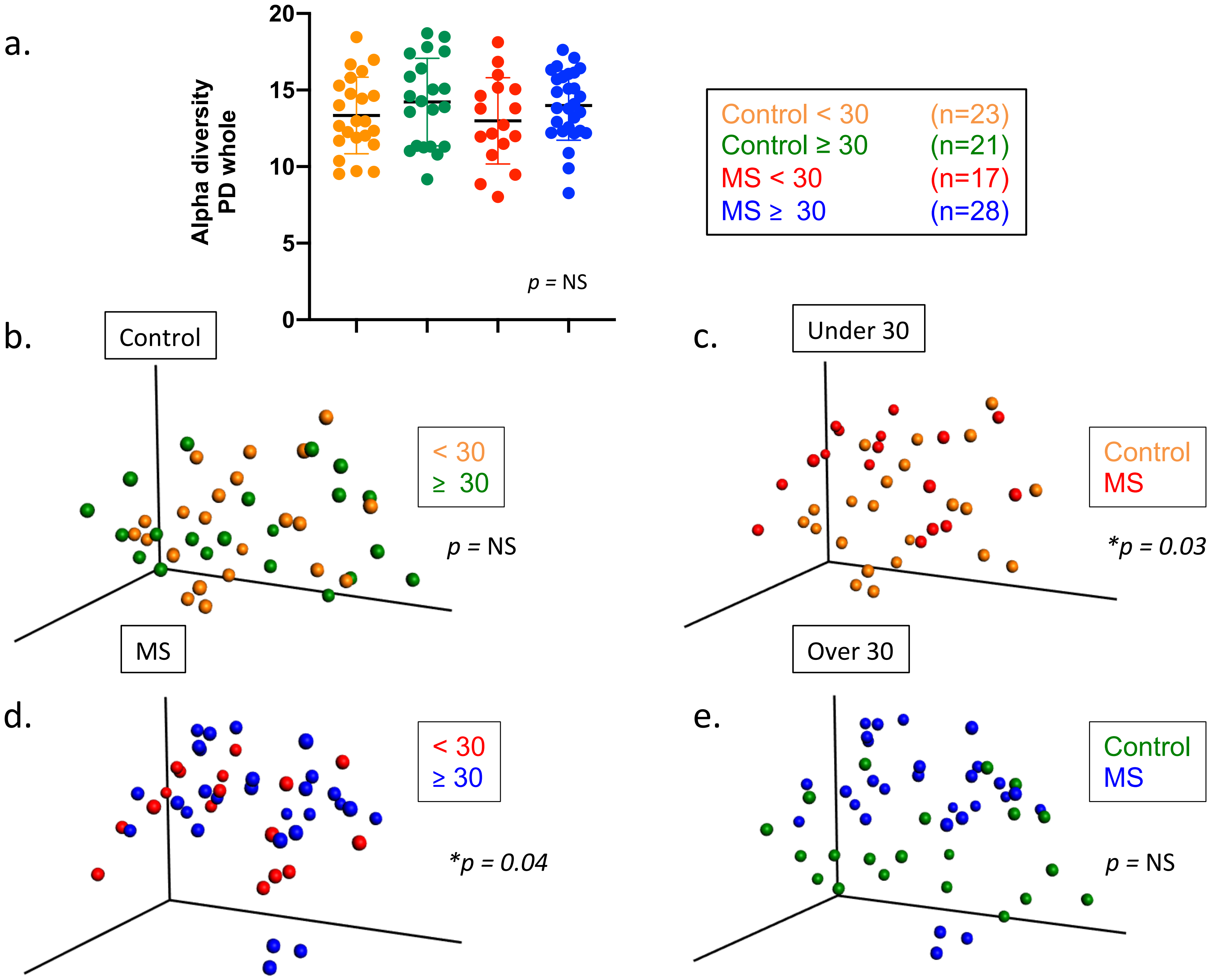

a.
Alpha diversity
PD whole
20
15
10
5
0
p = NS
Control < 30 (n=23)
Control ≥ 30 (n=21)
MS < 30 (n=17)
MS ≥ 30 (n=28)
b.
Control
< 30
≥ 30
p = NS
c.
Under 30
Control
MS
*p = 0.03
MS
d.
< 30
≥ 30
*p = 0.04
Over 30
e.
Control
MS
p = NS

Figure S2.

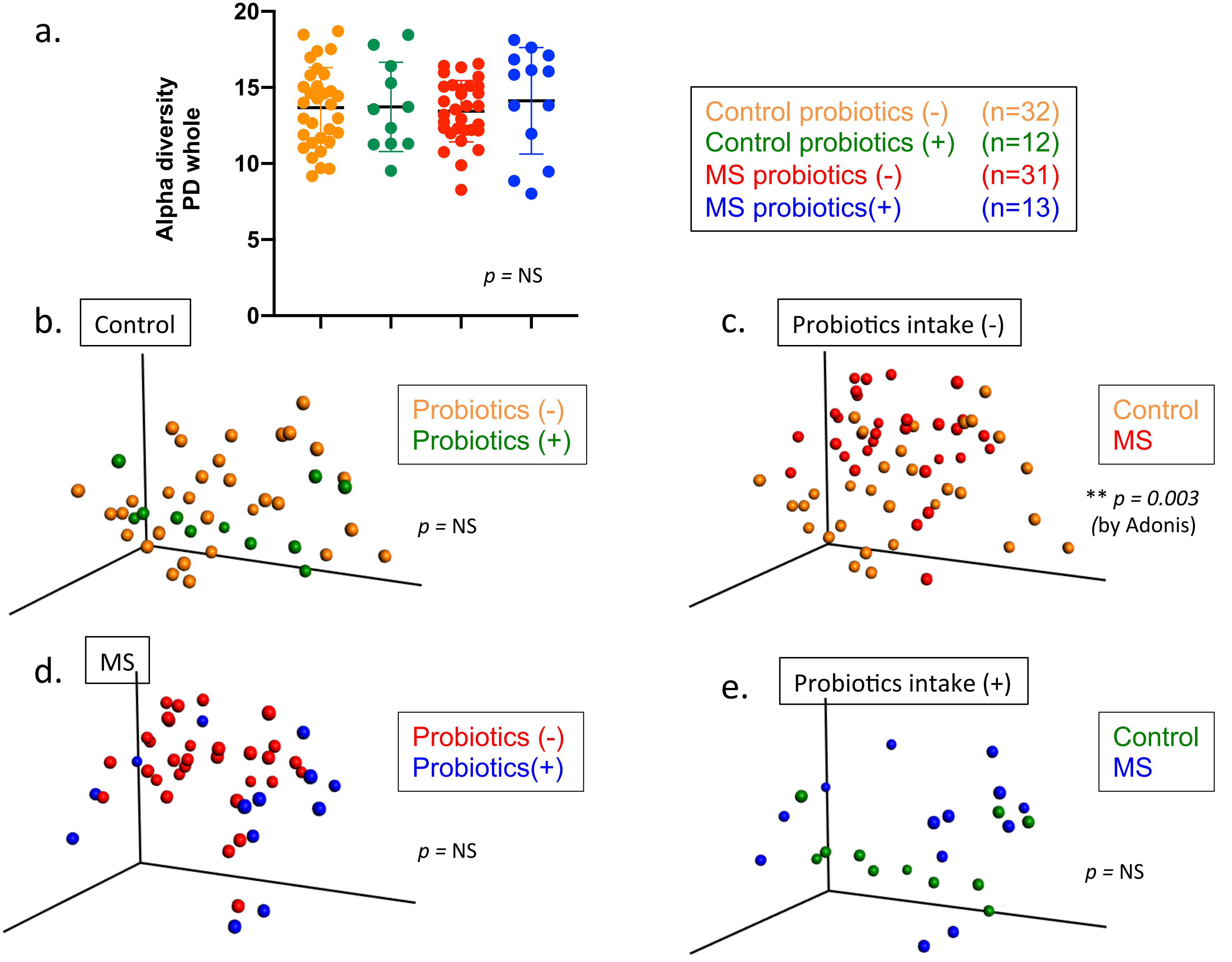
a.
Alpha diversity
PD whole
20
15
10
5
0
p = NS
Control probiotics (-) (n=32)
Control probiotics (+) (n=12)
MS probiotics (-) (n=31)
MS probiotics(+) (n=13)
b.
Control
Probiotics (-)
Probiotics (+)
p = NS
c.
Probiotics intake (-)
Control
MS
** p = 0.003
(by Adonis)
d.
MS
Probiotics (-)
Probiotics(+)
p = NS
e.
Probiotics intake (+)
Control
MS
p = NS

Figure S3.

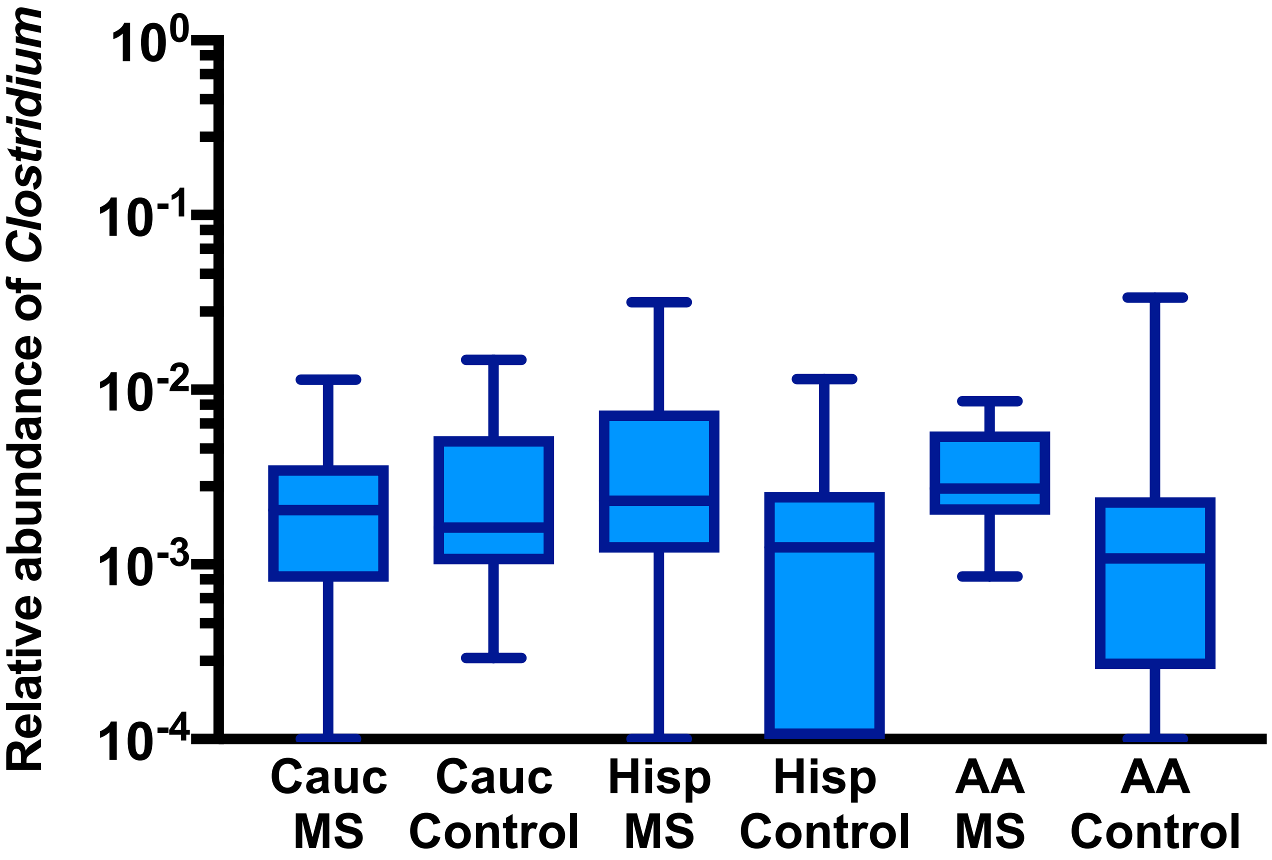

Relative abundance of *Clostridium*
10^0
10^-1
10^-2
10^-3
10^-4
Cauc MS
Cauc Control
Hisp MS
Hisp Control
AA MS
AA Control

Figure S4.
